# Refractory Hypocalcemia Following Total Thyroidectomy in an Adult Patient With Bariatric Surgery

**DOI:** 10.7759/cureus.69451

**Published:** 2024-09-15

**Authors:** Anna A Ilyasova, Christopher A Caulfield, Evan Raff

**Affiliations:** 1 Medicine, University of North Carolina at Chapel Hill School of Medicine, Chapel Hill, USA

**Keywords:** bariatric surgery complications, biliopancreatic diversion with duodenal switch (bpdds), hypocalcemia management, hypoparathyroidism, resistant hypocalcemia

## Abstract

Obesity poses a global health challenge with significant individual and societal impacts. Bariatric surgery, including Roux-en-Y gastric bypass (RYGB) and biliopancreatic diversion/duodenal switch (BPD/DS), is effective for long-term weight management but can lead to serious nutritional deficiencies, particularly hypocalcemia. This report presents the rare case of a 35-year-old woman with severe, recurrent hypocalcemia following BPD/DS surgery, complicated by iatrogenic hypoparathyroidism from prior thyroidectomy.

Despite aggressive oral and intravenous calcium and vitamin D supplementation, the patient's hypocalcemia remained refractory, necessitating multiple hospitalizations. Laboratory studies confirmed severe hypocalcemia, low parathyroid hormone (PTH), and deficiencies in fat-soluble vitamins, complicating her clinical management.

As conventional treatments failed, the patient underwent surgical revision from BPD/DS to RYGB anatomy, aimed at improving calcium absorption by restoring functional small bowel length. Postoperatively, her serum calcium levels normalized, and she was successfully discharged on oral calcium supplementation, with stable calcium levels at follow-up.

This case underscores the challenges of managing hypocalcemia in patients with BPD/DS anatomy and hypoparathyroidism. The greater malabsorption associated with BPD/DS can severely impair calcium absorption, leading to refractory hypocalcemia. This report is the first documented case where surgical conversion from BPD/DS to RYGB effectively treated this condition.

The findings underscore the critical need for preoperative risk assessment for, and careful postoperative management of, hypoparathyroidism in bariatric surgery patients with complex medical histories. This case report outlines a potential treatment pathway for managing refractory hypocalcemia, emphasizing the importance of preserving calcium-absorbing bowel function in patients with BPD/DS anatomy. It provides valuable insights into treating severe hypocalcemia and demonstrates a successful surgical intervention that could inform the management of similar cases.

## Introduction

Obesity, defined as a body mass index (BMI) of 30 kg/m² or higher, is a prevalent condition affecting people of all ages. The World Health Organization estimates that in 2022, approximately 890 million adults, 160 million children and adolescents, and 37 million children under five years old were affected by obesity globally [1,2]. In the United States, over two in five adults and one in five children have obesity [1,3]. The health and economic consequences of obesity are well documented [1]. Obesity is linked to the development of type 2 diabetes mellitus, heart disease, stroke, cancer, osteoarthritis, and obstructive sleep apnea, among others. In the United States, the estimated economic impact of obesity-related medical care was $173 billion in 2019 [4].

Bariatric surgery is a common weight loss strategy, with over 270,000 procedures performed in the United States in 2022 alone [5]. These surgeries not only aid in long-term weight management but also reduce the risk of obesity-related complications. Bariatric surgery is indicated for adults with a BMI ≥35 kg/m² or those with a BMI between 30 and 34.9 kg/m² who have type 2 diabetes or cannot achieve substantial, durable weight loss and comorbidity improvement through non-surgical methods. Two common bariatric surgeries are Roux-en-Y gastric bypass (RYGB) and biliopancreatic diversion/duodenal switch (BPD/DS) bypass. While these procedures are highly effective for achieving sustained weight loss, they can lead to significant nutritional deficiencies; therefore, the preoperative evaluation must include a consultation with a nutritionist to review the individual's weight history and eating behaviors, evaluate body composition and energy needs, and identify any micronutrient deficiencies. Among these deficiencies, hypocalcemia is a particularly serious concern. The risk of hypocalcemia arises due to alterations in the digestive system that can impair calcium absorption, necessitating careful monitoring and management to prevent adverse health outcomes. The prevalence of hypocalcemia after BPD/DS ranges from 7.3% to 48%, which is significantly higher compared to 0.9-3.6% observed following RYGB [6-10]. It is for this reason that postoperative nutrient assessments are recommended every 3-6 months during the first year after bariatric surgery and annually thereafter. 

This report presents the rare case of a 35-year-old woman with severe, recurrent hypocalcemia, resistant to medical therapy, in the context of iatrogenic hypoparathyroidism and BPD/DS anatomy. Conversion of her BPD/DS to RYGB resolved her hypocalcemia, providing valuable insights into managing such complex cases.

## Case presentation

A 35-year-old female with iatrogenic hypothyroidism and hypoparathyroidism and asthma was directly admitted from her outpatient infusion clinic following one day of progressive bone pain, bilateral hand spasms, and paresthesia of the extremities. She had undergone a thyroidectomy for a multinodular goiter two years prior, resulting in hypothyroidism and hypoparathyroidism. She also had a history of BPD/DS surgery four years prior (for a preoperative BMI of 55 kg/m^2^ and poorly controlled type 2 diabetes mellitus), complicated by severe postoperative hypocalcemia requiring aggressive calcium supplementation. In the year preceding this admission, she had been hospitalized twice for severe symptomatic hypocalcemia. Her medications included albuterol, fluticasone propionate/salmeterol, calcitriol, calcium carbonate, calcium gluconate, ergocalciferol, levothyroxine, and liothyronine.

On admission, she was afebrile with normal vitals. Physical examination revealed a positive Chvostek sign (unilateral twitching of the cheek and upper lip with tapping of the ipsilateral facial nerve) and left eyelid myoclonus. Initial serum studies showed hypocalcemia, hyperphosphatemia, low parathyroid hormone (PTH), low vitamin A, and low zinc, with otherwise normal creatinine, albumin, magnesium, potassium, 25-hydroxy vitamin D, and vitamin E and K levels (see Table 1).

**Table 1:** Abnormal patient laboratory results.

Laboratory	Patient result	Reference range
Serum calcium	6.5 mg/dL	8.7-10.4 mg/dL
Serum phosphorus	5.3 mg/dL	2.4-5.1 mg/dL
Serum parathyroid hormone	11.4 pg/mL	18.1-80.4 pg/mL
Serum vitamin A	28.8 mcg/dL	32.5-78 mcg/dL
Serum zinc	0.59 mcg/mL	60-106 mcg/mL

**Figure 1:**
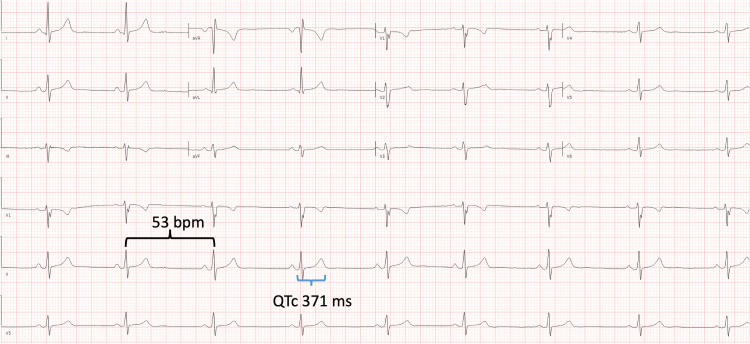
Patient ECG showing sinus bradycardia at a rate of 53 bpm (black bracket) and a QTc interval of 371 ms (blue bracket). ECG: electrocardiogram

Her electrocardiogram (ECG) showed sinus bradycardia at 53 bpm with a QTc of 371 ms (see Figure 1). Historical chart review revealed a 24-hour urine calcium level of <3 mg/dL during her hospitalization for hypocalcemia one month prior, suggesting significant gastrointestinal calcium malabsorption.

The patient was diagnosed with hypocalcemia syndrome due to combined iatrogenic hypoparathyroidism and malabsorption from BPD/DS anatomy. Despite increased dietary calcium intake and maximal dosing of oral calcitriol, calcium carbonate, ergocalciferol, and calcium citrate, she had daily recurrent severe hypocalcemia requiring intravenous calcium to achieve normal serum levels.

Surgical conversion from BPD/DS to RYGB was performed, resulting in the functional restoration of 300 cm of small bowel. Postoperatively, her serum calcium levels normalized, and she was discharged on only oral daily calcium supplementation. Her serum calcium remained stable at 8.7 mg/dL one month later.

## Discussion

**Figure 2:**
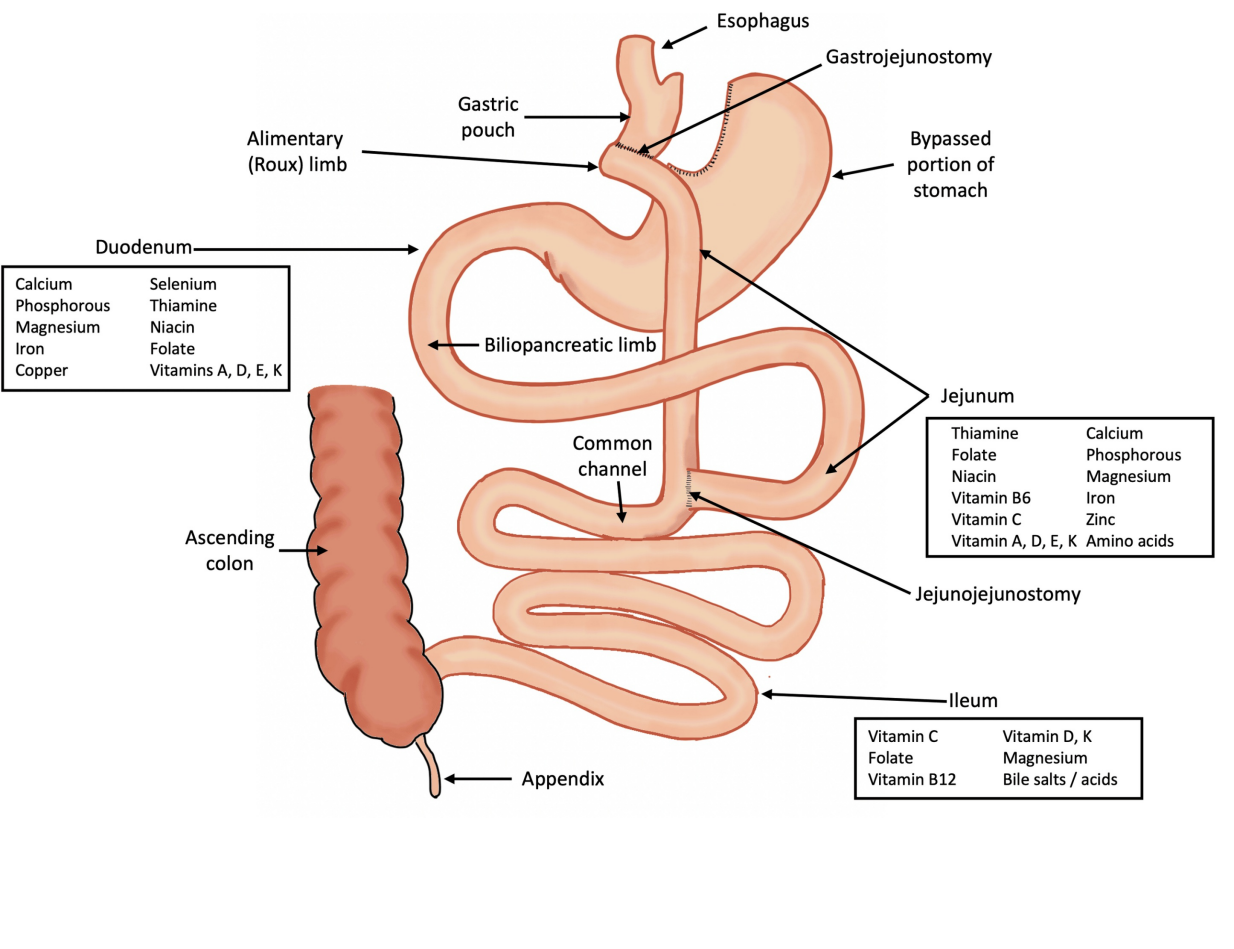
RYGB anatomy. RYGB: Roux-en-Y gastric bypass Image Credit: Authors

In RYGB, a small proximal gastric pouch is formed and anastomosed to a Roux limb of the jejunum, bypassing most of the stomach and duodenum (Figure 2). The remaining proximal small intestine, known as the biliopancreatic limb, is anastomosed to the Roux limb 75-150 cm distal to the gastrojejunostomy. This configuration restricts food intake and alters nutrient absorption by rerouting bile and pancreatic enzymes to mix with food in the more distal small intestine. RYGB is associated with risks of gastrointestinal hemorrhage, anastomotic leak, bowel obstruction, and, rarely, intussusception [11]. Although generally well controlled, nutritional deficiencies, especially iron deficiency, are seen in this partially malabsorptive anatomy [11].

**Figure 3:**
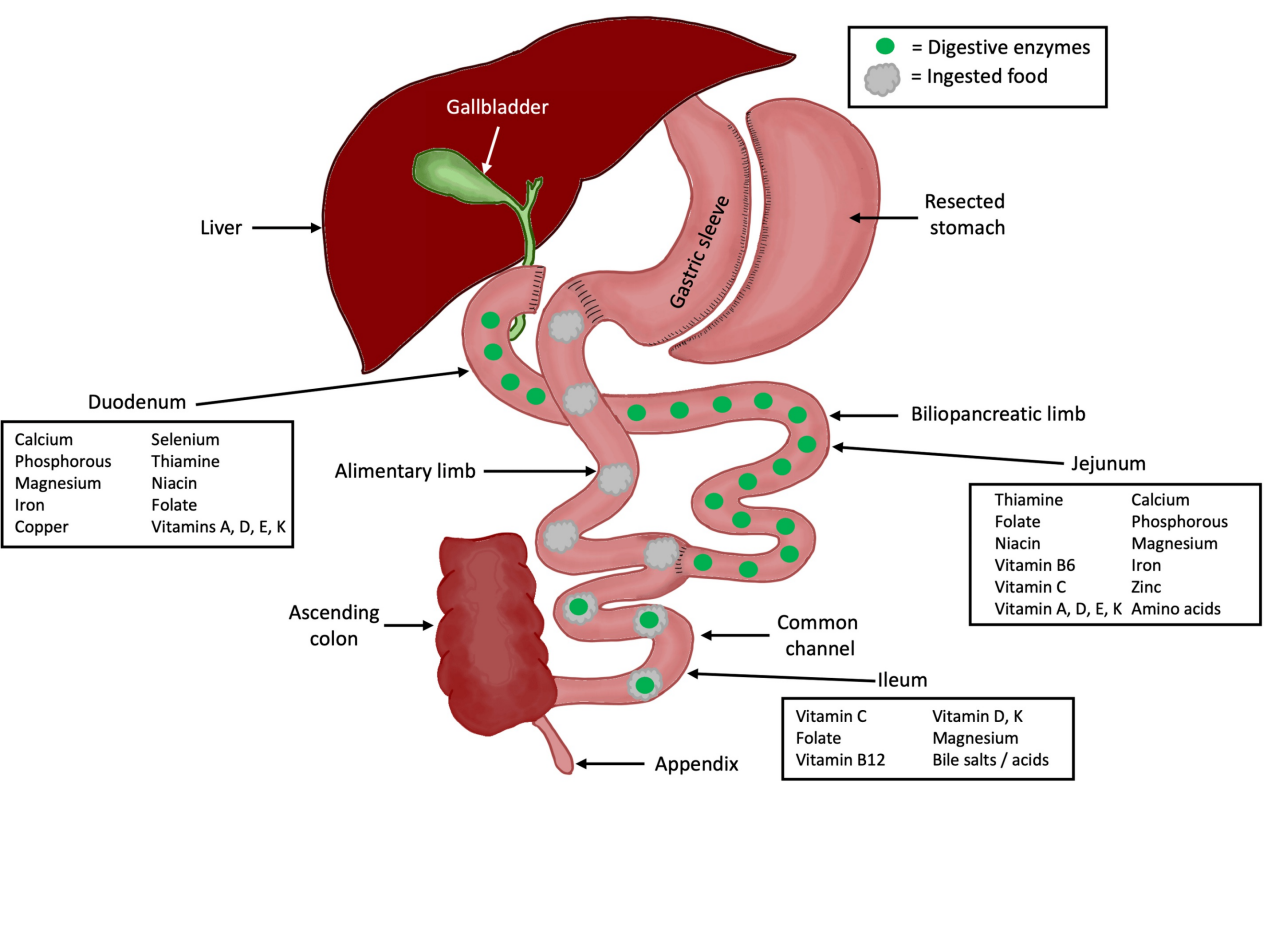
BPD/DS bypass anatomy. BPD/DS: biliopancreatic diversion/duodenal switch Image Credit: Authors

In BPD/DS bypass, a sleeve gastrectomy, in which most of the greater curvature of the stomach is removed and a tubular stomach is generated, is anastomosed to the proximal ileum (Figure 3). The remaining proximal biliopancreatic limb is anastomosed to the terminal ileum, 50-100 cm from the ileocecal valve. In this configuration, a significant bypass of the small intestine produces varying degrees of anatomical malabsorption [11,12]. The highly malabsorptive goal of BPD/DS surgery results in maximal weight loss outcomes, as well as the highest associated risks of fat-soluble vitamin (A, D, E, K), zinc, iron, selenium, magnesium, and calcium deficiencies [11].

Nutrient deficiency following bariatric surgery is a known complication. Decreased fat absorption may lead to fat-soluble vitamin deficiencies, as well as altered calcium metabolism [13]. As mentioned, the prevalence of hypocalcemia following BPD/DS ranges from 7.3% to 48%, higher than that of RYGB (0.9-3.6%) [6-10]. Differences in hypocalcemia risk between BPD/DS and RYGB are the result of varying small bowel surface area loss between the two surgeries. Most dietary calcium is absorbed by paracellular diffusion in the lower small intestine; thus, the additional small bowel bypassed in BPD/DS anatomy reduces calcium absorption in these patients [12,14]. 

**Figure 4:**
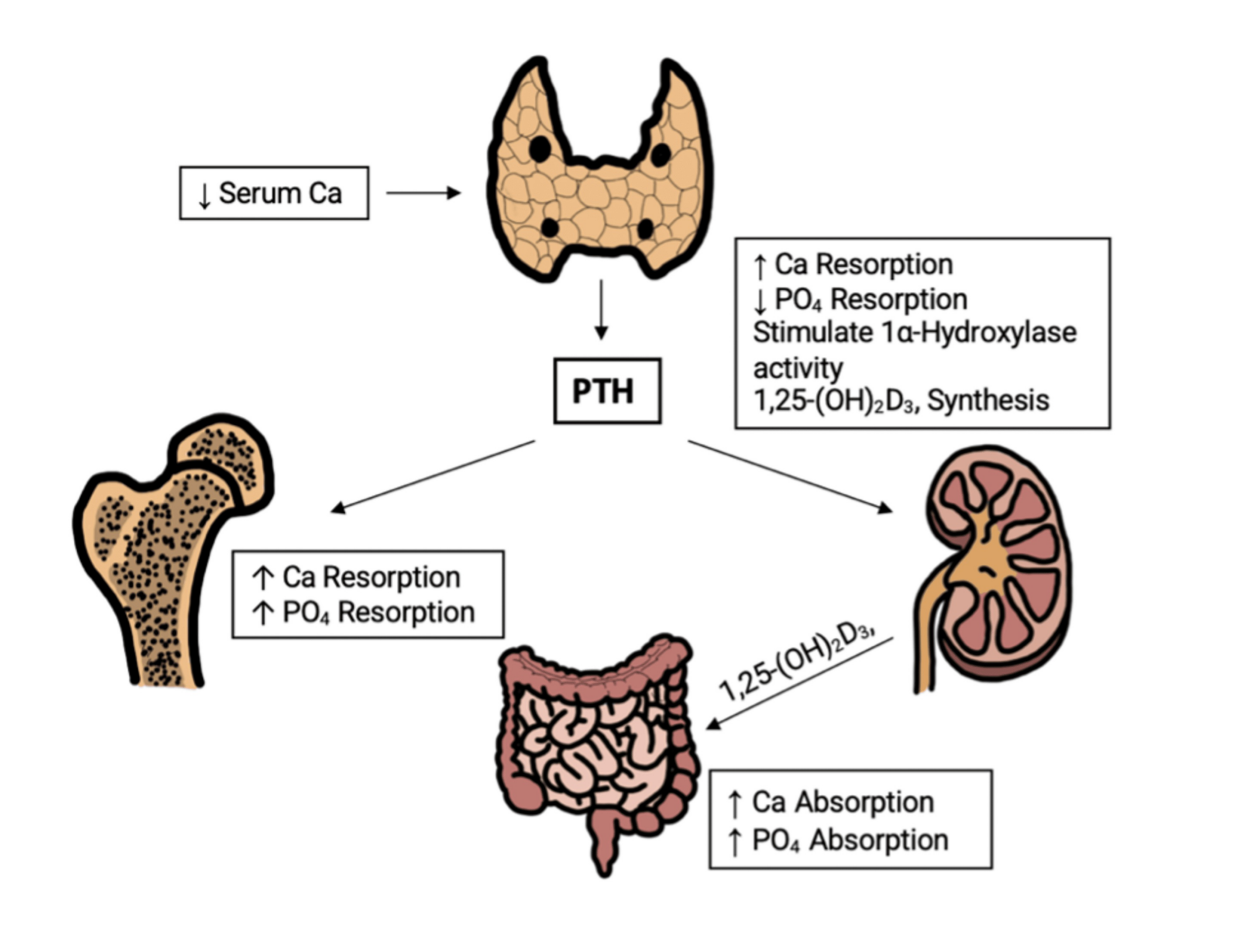
PTH and calcium homeostasis. PTH: parathyroid hormone Image Credit: Authors

Parathyroid glands play a crucial role in calcium homeostasis by releasing PTH, which functions to stimulate calcium release from bones, increase reabsorption in the kidneys, and enhance intestinal absorption through vitamin D activation (Figure 4) [15]. The release of PTH is triggered by hypocalcemia and plays a crucial role in compensating for calcium deficiency, particularly after gastric bypass surgery, where the anatomical changes reduce calcium absorption in the gut. Elevated PTH levels are seen in up to 68.6% and 29% of BPD/DS and RYGB patients, respectively [7,10].

Conversely, hypoparathyroidism may cause hypocalcemia in multiple ways. A lack of functional PTH, decreased renal responsiveness to PTH, or disorders of vitamin D metabolism, for example, can result in insufficient renal or small intestine calcium absorption [15]. Treatment of hypocalcemia in the setting of hypoparathyroidism includes oral and intravenous calcium and vitamin D supplementation and recombinant PTH when necessary [16]. In circumstances of gastric bypass anatomy, oral calcium and vitamin D supplementation may not be sufficient to maintain calcium levels due to reduced gastrointestinal absorption [17].

Current literature contains multiple examples of refractory hypocalcemia occurring after gastric bypass surgery and thyroidectomy [18,19]. Thorough pre-surgical assessment for hypoparathyroidism risk factors, such as a history of thyroid dysfunction, a family history of thyroid disease, or the presence of goiter, is crucial for patients being considered for gastric bypass surgery. Patients with preoperative renal impairment and postoperative vitamin D deficiency are most at risk for hypocalcemia following gastric bypass [6]. Patients with hypocalcemia resulting from gastric bypass and hypoparathyroidism have been effectively treated using oral vitamin D and calcium supplements, intravenous calcium gluconate infusions, calcium administered via gastrostomy, and, in rare instances, pancreatic lipase therapy [20]. Patients with severe hypocalcemia and hypoparathyroidism in the setting of RYGB anatomy have also been successfully treated with RYGB reversal [21]. For some patients, like the one presented in this case, weight regain following full bariatric surgery reversal may not be conducive to health goals. In this patient, her BMI prior to BPD/DS surgery was 55 kg/m^2^, reduced to 37 kg/m^2 ^at the time of this presentation, and increased to 43 kg/m^2 ^two years following BPD/DS conversion to RYGB.

## Conclusions

This case report describes a unique instance of refractory hypocalcemia in a patient with iatrogenic hypoparathyroidism and BPD/DS anatomy. It underscores the need for careful consideration of bariatric surgical options in patients with pre-existing conditions that may exacerbate nutrient deficiencies. While hypocalcemia and other nutrient deficiencies following BPD/DS are known, the severity of refractory hypocalcemia in this case is noteworthy. The successful surgical conversion from BPD/DS to RYGB provides a potential treatment approach for similar cases, prioritizing the preservation of calcium-absorbing bowel function while addressing the patient's clinical needs.
